# Radioimmunotherapy of B-Cell Non-Hodgkin’s Lymphoma

**DOI:** 10.3389/fonc.2013.00177

**Published:** 2013-07-11

**Authors:** Caroline Bodet-Milin, Ludovic Ferrer, Amandine Pallardy, Thomas Eugène, Aurore Rauscher, Alain Faivre-Chauvet, Jacques Barbet, Françoise Kraeber-Bodéré

**Affiliations:** ^1^Nuclear Medicine Department, University Hospital, Nantes, France; ^2^CRCNA, INSERM U892, CNRS UMR 7299, Université de Nantes, IRS-UN, Nantes, France; ^3^Nuclear Medicine Department, ICO-Gauducheau, Saint-Herblain, France; ^4^GIP ARRONAX, Saint-Herblain, France

**Keywords:** radioimmunotherapy, monoclonal antibody, CD20, CD22, dosimetry

## Abstract

This manuscript reviews current advances in the use of radioimmunotherapy (RIT) for the treatment of B-cell non-Hodgkin’s lymphoma (NHL). RIT has been in use for more than 20 years and has progressed significantly with the discovery of new molecular targets, the development of new stable chelates, the humanization of monoclonal antibodies (MAbs), and the use of pretargeting techniques. Today, two products targeting the CD20 antigen are approved: ^131^I-tositumomab (Bexxar^®^), and ^90^Y-ibritumomab tiuxetan (Zevalin^®^). ^131^I-tositumomab is available in the United States, and ^90^Y-ibritumumab tiuxetan in Europe, the United States, Asia, and Africa. RIT can be integrated in clinical practice using non-ablative activities for treatment of patients with relapsed or refractory follicular lymphoma (FL) or as consolidation after induction chemotherapy in front-line treatment in FL patients. Despite the lack of phase III studies to clearly define the efficacy of RIT in the management of B lymphoma in the era of rituximab-based therapy, RIT efficacy in NHL has been demonstrated. In relapsing refractory FL and transformed NHL, RIT as a monotherapy induces around 30% complete response with a possibility of durable remissions. RIT consolidation after induction therapy significantly improves the quality of the response. Dose-limiting toxicity of RIT is hematological, depending on bone marrow involvement and prior treatment. Non-hematological toxicity is generally low. Different studies have been published assessing innovative protocols of RIT or new indications, in particular treatment in patients with aggressive lymphomas. High-dose treatment, RIT as consolidation after different therapeutic induction modalities, RIT in first-line treatment or fractionated RIT showed promising results. New MAbs, in particular humanized MAbs, or combinations of naked and radiolabeled MAbs, also appear promising. Personalized dosimetry protocols should be developed to determine injected activity.

## Introduction

B-cell NHL can be classified into more than 25 histological subtypes according to World Health Organization (WHO) classification, and can be separated into aggressive (65% of NHL) and indolent forms (35%) (1). The Ann Arbor classification is used for staging, including 1–4 stages. Diffuse large B-cell NHL (DLBCL) is the most common type of aggressive NHL (31%), and follicular lymphoma (FL) the most common type of indolent NLH (22%). FL generally shows indolent progression with response to chemotherapy, but always relapses. Survival ranges from 5 to 15 years, depending on the Follicular Lymphoma International Prognostic Index (FLIPI) or Groupe d’Etude des Lymphomes Folliculaires (GELF) prognosis score. Prognosis of DLBCL is different, with 50–60% of patients being cured, with prognosis depending on the International Prognostic Index (IPI) score. Mantle cell lymphoma (MCL) represents 6% of NHL and has a poor overall survival (OS), with a median survival of about 5 years in young patients, but often lower survival rates in elderly patients. Treatment of disseminated NHL includes multi-agent chemotherapy, with the possibility of high-dose (HD) chemotherapy coupled with stem-cell transplantation (SCT) in high-risk young people (<60–65 years). In patients with aggressive or indolent B-NHL, the introduction of rituximab (Mabthera^®^, Rituxan^®^, Roche Ltd., Genentech, Basel, Switzerland), a monoclonal chimeric anti-CD20 antibody, when combined with different chemotherapy regimens (R-chemotherapy) resulted in an improvement in patient outcome, compared with chemotherapy alone (2–4). Response rates with rituximab alone is rather modest (5). Involved-field radiation can be proposed for limited stage FL or treatment of residual masses of DLBCL.

Radioimmunotherapy (RIT) is a targeted therapy whereby irradiation from radionuclides is delivered to tumor targets using monoclonal antibodies (MAbs) directed to a tumor antigen (6, 7). NHL cells express well-characterized antigens, are highly radiosensitive, respond to cold mAbs, and represent a relevant indication for RIT. In 1988, DeNardo et al. reported the first RIT clinical trial in resistant NHL, using the anti-HLA-DR Lym-1 MAb labeled with iodine-131 (8). Over the last 20 years, RIT has significantly progressed with the development of new humanized mAbs, stable chelates for labeling, and pretargeting techniques (9). The cytotoxic mechanisms of RIT involve both radiobiological and immunological processes (10). MAbs, particularly rituximab, may exert cytotoxic effects through apoptosis, antibody-dependent cell-mediated cytotoxicity (ADCC), and complement-dependent cytotoxicity (CDC). When MAbs are labeled with radionuclides, the combination of immunological and radiobiological cytotoxicity, including bystander effects, results in higher anti-tumor efficacy (10). RIT delivers continuous heterogeneous low-dose-rate irradiation (<1 Gy/h) concomitant with biological effects such as the repair of radiation-induced sublethal damage (10). Fowler evaluated the effects of low-dose-rate on the relative effectiveness of radiation as a function of the repair capacities of tissues using a mathematical model (11). They conclude that DNA repair lowers the efficacy of low-dose-rate irradiation, but that this effect may be overcome in part by cell cycle arrest in the G2/M phase, which makes cells more radiosensitive. However this hypothesis is now challenged by other explanations such as a decreased sensing of DNA damage by ATM at low-dose-rate that results in decreased activation of the early DNA damage response and repair (12). Other effect such as reoxygenation of hypoxic cells during the irradiation and effects on tumor vessels have been proposed to explain the relatively high efficacy of RIT (10). It is most likely that a combination of these effect and additional biological and immunological mechanisms are at work to make RIT effective in lymphoma, in spite of the low-dose-rate and low absorbed dose delivered by the therapeutic approach.

Although a dose-effect relationship has not yet been clearly demonstrated, it is likely to be present even if such a relationship could be masked by the anti-tumor effects of cold MAbs that are generally injected prior to the radiolabeled antibody. The choice of appropriate antibodies and radionuclide is critical (13). The path-length of penetration of the radioactive emission should match the size of the targeted tumor. Yttrium-90, with its long-range beta emission, is better suited for bulky disease. However, promising results have been observed using ^90^Y-RIT in the consolidation setting in patients in partial response (PR) or complete response (CR) after induction therapy (14). Radionuclides such as ^131^I or ^177^Lu with shorter-range energy emissions should be more favorable in the setting of minimal residual disease (MRD). In this MRD clinical setting, biodistribution and tumor dosimetry are more favorable, tumor cells are less hypoxic and more radiosensitive (15), and immunotherapy is more efficient (16).

Today, two products targeting CD20 have been approved: ^131^I-tositumomab (Bexxar^®^; GlaxoSmithKline), and ^90^Y-ibritumomab tiuxetan (Zevalin^®^, Spectrum Pharmaceuticals, Henderson, NV, USA). ^131^I-tositumomab is available in the United States, and ^90^Y-ibritumumab is approved in Europe, the United States, Asia, and Africa. RIT can be integrated in clinical practice using non-ablative activities for treatment of patients with relapsed or refractory FL or as consolidation after induction chemotherapy in front-line treatment in FL patients. Different RIT protocols are assessed in clinical trials in FL or other lymphoma subtypes: myeloablative or HD treatment, RIT as consolidation after chemotherapy to target MRD, RIT in first-line treatment, fractionated RIT and other MAbs especially targeting antigens other than CD20. Moreover, personalized dosimetry protocols are proposed to better predict dose-effect relationships.

## RIT of NHL in Clinical Practice

### Administration schemes

Bexxar^®^ and Zevalin^®^ are administered 6–8 days after a predose of cold MAb, respectively 2 × 450 mg of tositumomab and 2 × 250 mg of rituximab, to improve biodistribution and tumor targeting. The whole therapy requires only two outpatient visits. No dosimetry study is required for Zevalin^®^. When a dosimetry study is performed for research purposes, the first dose of cold MAb is injected with 5 mCi of ^111^In-ibritumomab tiuxetan. The injected activity depends on body weight and platelet count. The therapeutic dose is 0.4 mCi/kg (14.8 MBq/kg) (0.3 mCi/kg, 11.1 MBq/kg, in patients with a platelet count of 100,000–149,000/mm^3^) to a maximum total activity of 32 mCi (1,184 MBq) (17). Bexxar^®^ is delivered after a dosimetry study to identify patients whose biodistribution profiles preclude administration of the therapeutic step, and to adapt therapeutic activity to whole-body clearance of the radiolabeled MAb. Whole-body clearance is determined by a series of three scans recorded after infusion of 5 mCi of ^131^I-tositumomab. This dosimetry study allows determination of the injected therapeutic activity required to deliver a whole-body dose of 65–75 cGy. It has been demonstrated that if all patients had been injected with a standard dose of 40.7 MBq/kg, half of them would have been under or overdosed according to the whole-body clearance studies (18).

### Efficacy

Despite the lack of phase III studies to clearly define the role of RIT in the management of B lymphoma in the era of rituximab-based therapy, RIT efficacy in B lymphoma has been demonstrated, with the likelihood of providing a durable response. In a study of 143 patients with relapsed or refractory FL or transformed B-cell NHL, Zevalin^®^ appeared more efficient than rituximab, with the OR and CR rates significantly higher with Zevalin^®^ (80 vs. 56%, *p* = 0.002, and 30 vs. 16%, respectively, *p* = 0.04) (87). Patients refractory to rituximab had a 74% OR and those with thrombocytopenia 83% OR (87, 19). Mean time to progression (TTP) in responders was 12.6 months. OR was observed in 50% of patients with bulky lymphoma. Chemotherapy, administered in patients treated with RIT, was not associated with higher toxicity (20). In a meta-analysis involving relapsed NHL patients treated with Zevalin^®^ in four clinical trials, long-term responses (TTP > 12 months) were seen in 37% of patients (21). At a median follow-up time of 53.5 months, the median TTP was 29.3 months. A third of these patients had been treated with at least three previous therapies, and 37% of them had not responded to their last therapy. The estimated 5 year-OS was 53% for all patients treated with Zevalin^®^ and 81% for long-term responders.

Using Bexxar^®^, a long-term meta-analysis performed on 250 heavily pre-treated patients with indolent lymphoma treated in five clinical trials, OR rates ranged from 47 to 68% and CR rates from 20 to 38% (22). For the durable response population, the median duration of response was 45.8 months and the median duration of response had not been reached at 5 years for those who had achieved a CR. Interestingly, patients who showed durable responses had poor prognostic characteristics (bone marrow involvement in 41%, bulky disease ≥5 cm in 49%, and transformed histology in 23%).

In 2011 The International Radioimmunotherapy Network (RIT-N) reported on the long-term observational data from 467 Zevalin^®^ -treated patients with an observation time of at least 12 months outside the randomized clinical studies (23). Lymphoma subtype was documented for all patients at initial diagnosis: 58% FL, 20% DLBCL, 14% MCL, and less than 10% other subtypes. Most patients had stage IV disease according to the Ann Arbor Classification, and 15% had bone marrow infiltration. FL patients received RIT as consolidation in 45% of cases, after first- and second-line therapy, and for recurrence in 37.7% of cases, in second- and more than third-line therapy. For patients with other lymphoma subtypes, RIT was predominantly used as consolidation (69%) in first-line therapy and in recurrence (13%) in second- and more than third-line therapy. Response was documented for 448 patients (260 with FL and 188 with other lymphoma subtypes). Three hundred and thirty-six (75%) patients achieved a CR and 70 (16%) a partial remission (PR). The CR rate in FL patients was 73%, as compared with 77% in other lymphoma subtypes. The difference in CR rates and progression-free survival (PFS) between the pivotal studies and the RIT-N study could be explained by a different patient selection, with treatment of patients earlier in clinical practice than in pivotal studies. Previously, Emmanouilides et al. showed that earlier injection of Zevalin^®^ lead to better outcomes (24). As compared to patients treated at least at second relapse, patients treated at first relapse had a higher OR rate (86 vs. 72%, *p* = 0.051) and CR rate (49 vs. 28%, *p* = 0.004) and longer TTP (12.6 vs. 7.9 months, *p* = 0.038). Similarly, higher OR and CR rates were observed in patients treated by Bexxar^®^ at first or second relapse (25).

In an interesting recent review on treatment of lymphoma by RIT, Illidge regretted the low implementation of RIT in current clinical practice (26). Despite the unique non-cross-reactive mechanisms of action of RIT with proven high clinical efficacy in patients resistant to chemotherapy and rituximab (probably the most active approaches ever developed for NHL), RIT has failed to be adopted by hemato-oncologists. Both ^131^I-tositumomab and ^90^Y-ibritumomab have demonstrated high clinical efficacy in heavily pre-treated populations, including patients with disease refractory to both chemotherapy and rituximab underlying their unique mechanisms of action of RIT.

### Safety

Hematological toxicity is the major side effect of RIT, and depends on bone marrow involvement and prior treatment (27–29). After a 0.4 mCi/kg of Zevalin^®^, grade-4 neutropenia, thrombocytopenia, and anemia occurred in 30, 10, and 3% of patients, respectively. Using Bexxar^®^, grade-4 neutropenia, thrombopenia, and anemia were observed in 17, 3, and 2%, respectively. Nadir occurred 7–9 weeks after Zevalin^®^ injection, and 4–6 weeks after Bexxar^®^ injection. In the long-term analysis of the international RIT-N, no significant difference was observed between patients with FL and patients with other lymphoma histologies (23). Time to complete recovery of blood count (hemoglobin > 12 g/dL, platelets > 150,000/μL, and leukocytes > 4,300/μL) had a median of 99 days for FL patients and 97 days for patients with other lymphoma subtypes. For hemoglobin, the nadir was reached in a median of 47.5 days for FL and 42 days for patients with other lymphoma subtypes, for platelets, in a median 35 days for FL and other lymphoma subtypes and for leukocytes, in a median of 40 days for FL and 38 days for patients with other lymphoma subtypes.

Non-hematological toxicity is generally low, including asthenia, anorexia, fever, nausea, headache, chills, arthralgia, and myalgia. Allergic reactions have been observed during MAb infusion, in particular after the first rituximab injection before Zevalin^®^. It is important to highlight that RIT is well tolerated by older patients and represents a strong treatment choice in this group of patients. Immunogenicity with human anti-mouse and human anti-chimeric antibody production was observed, ranging from 1 to 63% between studies. The immunogenicity risk was significantly higher in previously untreated patients (30).

Secondary myelodysplastic syndrome (MDS) or acute myelogenous leukemia were reported in 1–3% of cases (23, 27, 28, 31). The risk appears to be increased in patients previously treated by several lines of chemotherapy or radiotherapy. However, no causal relationship between RIT and subsequent MDS has been established, and there is ongoing debate about the role of prior therapy (i.e., fludarabine) as the causative agent rather than RIT itself (31). Moreover, prior RIT has not posed a limitation to subsequent stem-cell collection and transplantation (20, 32). A cytological and genetic analysis of bone marrow could be proposed for heavily pre-treated patients prior to beginning RIT. In the long-term analysis of the international RIT-N, the rate of secondary solid tumors was 0.8%, including breast cancer, prostate cancer, multiform glioblastoma, and non-small-cell lung cancer (23).

Finally, it is important to highlight that toxicity due to RIT is relatively low compared with the side-effects of anthracycline-based combination chemotherapy that includes significant early toxicity with vomiting, alopecia, neutropenia, infection, neuropathy, and late toxicity with cardiomyopathy, neuropathy.

## High-Dose Treatment

These approaches require autologous or allogeneic SCT and consist of injecting RIT myeloablative activity or a combination of standard or escalated activity of RIT with HD chemotherapy. The rationale of HD approaches is to deliver curative radiation doses to tumor sites while limiting exposure to normal organs. The rationale of RIT combined with chemotherapy is to obtain a synergistic effect between radio-sensitizing chemotherapy and radiation.

### HD RIT

Administration of HD RIT without chemotherapy could be beneficial in aggressive NHL, which probably requires higher absorbed doses than indolent NHL, and for patients older than 60 years, which are often denied potentially curative HD chemotherapy because of the risk of excessive treatment-related morbidity and mortality. Liu et al. reported results of RIT using activities of 10.4–29.0 GBq (280–785 mCi) of ^131^I-tositumomab in 29 patients with relapsed B-cell NHL (33). The OR was 86%, with 79% CR and, with a median follow-up of 42 months, the estimated OS and PFS rates 68 and 42%, respectively. The non-hematopoietic dose-limiting toxicity (DLT) was reversible cardiopulmonary insufficiency, which occurred in two patients at doses ≥27 Gy to the lungs. Late side-effects included renal insufficiency in one patient, and functional cardiac impairment in another. Two patients developed second malignancies, but none have developed MDS. In 2007, Gopal et al. reported their experience with HD ^131^I-tositumomab activity in 24 patients older than 60 years with relapsed B-cell NHL (34). About 12–42.7 GBq (328–1,154 mCi) were injected to deliver 25–27 Gy to the critical normal organ receiving the highest radiation dose. Autologous SCT was performed approximately 2 weeks after therapy. The estimated 3 year OS and PFS rates were 59 and 51%, respectively, with a median follow-up of 2.9 years. There were no treatment-related deaths, and only two patients experienced grade-4 non-hematological toxicity.

Feasibility of HD Zevalin^®^ (0.8–1.5 mCi/kg, 57–150 mCi, 2.1–5.55 GBq) was also reported in 13 refractory NHL patients (eight DLBCL, one FL, three MCL, and one transformed MZL) (35). Median age was 68 years (28–73), with a median of three prior therapy courses including HD chemotherapy (1–6). The dosimetry study showed acceptable calculated absorbed doses to normal organs. Two patients were treated at a lower activity level because of elevated liver uptake. Infections and liver toxicity were observed, but no pulmonary, cardiac or renal toxicity. About 8 of 13 patients showed objective response with six CR and two PR. One patient still in CR for MCL developed a MDS 2 years after treatment. Thus, HD RIT could be considered as an effective treatment modality for resistant/refractory NHL considered unsuitable for aggressive salvage treatments.

### HD treatment combining RIT and chemotherapy

#### Regimens using escalated doses of RIT

Press et al. conducted a phase I/II trial to estimate the maximum tolerated dose (MTD) of ^131^I-tositumomab that could be combined with etoposide and cyclophosphamide, followed by autologous SCT, in 52 patients with relapsed B-cell NHL (36). The MTD of ^131^I-tositumomab that could be safely combined with 60 mg/kg etoposide and 100 mg/kg cyclophosphamide was calculated to deliver 25 Gy to critical normal organs. The estimated OS and PFS at 2 years was 83 and 68%, respectively. These findings compare favorably with those in a non-randomized control group of patients who underwent transplantation, external-beam total-body irradiation (TBR), and etoposide and cyclophosphamide therapy during the same period (OS of 53% and PFS of 36% at 2 years), even after adjustment for confounding variables in a multivariate analysis. Interestingly, survival was improved in aggressive and indolent NHL.

This approach was also validated in 16 patients with relapsed or refractory MCL (37). The enrolled patients had received a median of three prior treatments. The median activity of iodine-131 was 510 mCi (18.87 GBq). There were no therapy-related deaths. Among the 11 patients with conventionally measurable disease at the time of treatment, the respective CR and OR were 91 and 100%. Fifteen patients remained alive, and 12 without progression at 6–57 months after transplantation. OS at 3 years after transplantation was estimated at 93% and PFS at 61%.

Today, a standard HD schedule before transplantation includes carmustine, etoposide, cytarabine, and melphalan (BEAM). Winter et al. assessed a Z-BEAM regimen using escalated doses of Zevalin^®^ (900–1700 cGy to critical organs, 0.3–1.2 mCi/kg) in 44 patients with chemo-refractory NHL (55% DLBCL, 16% Richter, 16% MCL, 11% low-grade) (38). Two DLTs occurred at the 1700 cGy dose level. One heavily pre-treated patient developed MDS 291 days after the treatment. The 3-year OS and PFS rates were 52 and 37%, respectively. For dosimetry-based trials, 1500 cGy to the critical organ was the recommended dose (around 0.8 mCi/kg of Zevalin^®^). Outcomes were encouraging given the high-risk patient population.

#### Regimens using standard doses of RIT combined with high-dose chemotherapy

BEAM was also assessed when combined with standard dose of RIT. Vose et al. determined, in a phase I trial, the maximum outpatient dose of ^131^I-tositumomab (up to 0.75 Gy total-body dose) combined with BEAM followed by autologous SCT for the treatment of chemotherapy-resistant relapsed or refractory NHL (39). Twenty-three patients received 0.30–0.75 Gy total-body dose of RIT. The CR rate was 57% and the OR rate 65%. Short-term and long-term toxicities were similar to historical control patients treated with BEAM alone. With a median follow-up of 38 months (range, 27–60 months), OS was 55%, and event-free survival (EFS) 39%.

The combination of BEAM with standard dose of ^90^Y-RIT has also been assessed. Shimoni et al. reported the safety and outcome following standard-dose Zevalin^®^ (0.4 mCi/kg) followed by HD BEAM and autologous SCT in 23 patients (median age 55 years; ranging from 35 to 66) with chemo-refractory NHL (15 DLBCL, 7 Richter, 1 MCL), either primary refractory or in refractory relapse (40). Rituximab followed by Zevalin^®^ were given on day-14 and HD BEAM started on day-6. Of the 21 patients evaluated, 11 achieved CR and 9 achieved PR, 5 of whom converted to CR with additional radiation therapy (overall CR rate 76%). The estimated 2 year OS and PFS were 67 and 52%, respectively. The day-100 rate of treatment-related mortality was 9% (95% CI, 2–33%), and the 2-year cumulative incidence of relapse was 31% (95% CI, 17–57%). Extensive prior therapy (>3 lines), high LDH and IPI score at ASCT, bulky disease, and progression during last chemotherapy were risk factors for reduced survival.

Krishnan et al. conducted another study combining Zevalin^®^ with HD chemotherapy and autologous SCT using BEAM in patients with non-Hodgkin’s lymphoma and who were considered ineligible for TBR because of older age or prior radiotherapy (41). Eligible patients had CD20 positive refractory FL (4), poor-risk MCL (13), DLBCL (20), or transformed lymphoma (4). Median age was 60 years (range, 19–78 years). With a median follow-up of 18.4 months, the global estimated 2 year-overall OS was 88.9% (89.7% for DLBCL, 84.6 for MCL, and 85% for FL, respectively). Adverse events were similar to those seen historically with HD BEAM alone. More recently and in order to compare RIT and TBR-based conditioning regimens of ASCT, Krishnan et al. conducted a matched-cohort analysis in 92 DLBCL patients treated with either Z-BEAM (0.4 mCi/kg of Zevalin) or TBI-based conditioning regimens (fractionated TBI at 1200 cGy, with etoposide and cyclophosphamide) (42). OS at 4 years was 81.0% for the Z-BEAM and 52.7% for the TBI group (*p* = 0.01). There was no significant difference in the 4-year cumulative incidence of relapse/progression between Z-BEAM or TBI regimen (40.4 and 42.1%, respectively), whereas the non-relapse mortality was significantly higher in the TBI group (0% compared with 15.8% for TBI at 4 years), underlying the potentially lower toxicity of Z-BEAM.

In a recent prospective multicenter study, Shimoni et al. also demonstrated that standard-dose Zevalin^®^ (0.4 mCi/kg) combined with BEAM HD chemotherapy was safe and possibly more effective than BEAM alone as a conditioning regimen for ASCT in 43 patients with relapsed/refractory aggressive non-Hodgkin lymphoma (31 DLBCL, three mediastinal lymphomas and nine transformed FL) (43). There was no difference in engraftment kinetics between the two study arms. There was no significant added toxicity with the Z-BEAM regimen although there was a trend for more mucositis and more serious infections in this group. The 2-year PFS was 59 and 37% in the Z-BEAM and BEAM arms, and the 2-year OS was 91 and 62%, respectively. Interestingly, multivariate analysis identified BEAM alone as one of the poor prognostic factors. The results of this randomized study confirmed all the previously reported observations that the addition of Zevalin^®^ to HD chemotherapy is safe and not associated with excess toxicity. In order to definitively validate Z-BEAM regimen as a standard of care for ASCT, larger multicentric phase III clinical studies should be undertaken to better assess survival in homogenous groups of NHL patients, previously treated with rituximab-containing front-line and second-line chemotherapy.

#### Regimens using standard doses of RIT combined with allogeneic SCT

Recently, reduced-intensity conditioning (RIC) regimens have been developed to permit elderly patients or patients with co-morbidities, contraindicated for HD myeloablative chemotherapy, to allow allogeneic SCT. In order to increase the efficacy of the allogeneic graft, new strategies have been employed to increase the activity of RIC by adding Zevalin^®^ in the conditioning regimen. Bethge et al. designed a study to evaluate the feasibility of adding RIT to allo-SCT (44). Forty patients with low-grade advanced NHL were enrolled in this phase 2 study combining RIT using standard dose of Zevalin^®^ with RIC using fludarabine and 2 Gy TBI followed by allogeneic SCT. Combination of RIT with RIC seemed not to increase the toxicity in comparison to the previous experience in patients conditioned with fludarabine/2 Gy TBI alone. The study concluded that combined use of RIT with RIC was feasible with acceptable toxicity, even in elderly and heavily pre-treated patients. Another study performed by Gopal et al. also examined the combined use of RIT with RIC (fludarabine and 2 Gy TBR) for a non-myeloablative allo-SCT (45). Dosimetry was studied on day-21 before standard dose of Zevalin^®^. Forty patients were included: 18 with indolent lymphoma, 14 with DLBCL (7 *de novo* and 7 aggressive transformations), and 8 with MCL. At a median follow-up of 30 months, the estimated 2 year OS and PFS were 54 and 31%, respectively. Multivariate analysis revealed that patients with aggressive histology had poor OS and PFS when compared with indolent histology (*p* < 0.01). This prospective phase II trial concluded that the combined use of RIT with RIC was safe and feasible and was able to induce objective remissions in the majority of these high-risk patients, which were otherwise not previously considered candidates for either standard myeloablative or non-myeloablative transplantations, including patients with chemo-resistant, bulky disease, or aggressive histology.

## RIT as Consolidation after Induction Therapy

In the RIT-N analysis, a high efficacy in both FL and other lymphoma subtypes was observed when RIT was applied as part of the first-line treatment, as consolidation after induction therapy, to target MRD (23). The FIT randomized phase III trial showed the benefits of Zevalin^®^ as consolidation in previously untreated FL patients (14). After completing induction therapy, patients were randomized to receive either standard dose of Zevalin^®^ (*n* = 208) or no further treatment (*n* = 206). Induction therapies included CVP/COP (*n* = 106), CHOP and CHOP-like (*n* = 183), fludarabine combinations (*n* = 22), chlorambucil (*n* = 39), and rituximab-chemotherapy combinations (*n* = 59). A high conversion rate from PR to CR of 77% was observed after RIT, leading to a high CR rate of 87% after RIT. Interestingly, the same CR rate was obtained after RIT in all subgroups of induction therapy, despite the difference in CR rate between the initial chemotherapy regimens. The quality of the response improvement was associated with increase of PFS of more than 2 years in the RIT-consolidation arm as compared to the control arm. However, no significant increase of PFS was observed in the sub-group of patients receiving a rituximab-based therapy as induction, probably because of the statistically small number of patients treated with this regimen. RIT could be considered as an alternative to rituximab for combination with CHOP. The Southwest Oncology Group (SWOG) and Cancer and Leukemia Group B recently reported the results of the phase III randomized intergroup protocol (SWOG S0016) that enrolled 554 patients with previously untreated, advanced-stage FL to compare six cycles of R-CHOP at 3 week intervals with six cycles of CHOP followed by consolidation with tositumomab/iodine I-131 tositumomab (46). However, no benefits were observed in the RIT arm: after a median follow-up period of 4.9 years, the 2-year estimated PFS was 76% on the CHOP-R arm and 80% on the CHOP-RIT arm (*p* = 0.11), and the 2-year estimated OS 97% on the CHOP-R arm and 93% on the CHOP-RIT arm (*p* = 0.08).

The fact that no benefit of RIT has been demonstrated as an alternative to rituximab combined with CHOP, or as consolidation after 6–8 cycles of R-CHOP, constitutes a limit in the development of RIT in an era in which R-CHOP has substantially improved outcome and represented a therapeutic standard. Moreover, rituximab maintenance treatment after R-chemotherapy was recently demonstrated to improve the relapse-free survival in a large phase III study (47). Several reports suggested comparable efficacy of RIT consolidation and rituximab maintenance; however these two approaches have not been compared in a randomized trial. Ideally, rituximab and RIT should be considered as complementary approaches with possible additive or synergistic effect. This could be achieved by performing a, randomized phase III trials to compare maintenance by rituximab versus consolidation by RIT or maintenance by rituximab versus consolidation by RIT+ maintenance by rituximab, following induction with R-CHOP.

The use of RIT as consolidation might also allow reduced chemotherapy cycle number. Leonard et al. reported in 2005, in 35 previously untreated FL patients, the efficacy of three abbreviated courses of fludarabine followed by, 6–8 weeks later, tositumomab and ^131^I-tositumomab (48). After fludarabine, 31 (89%) of 35 patients responded, with three (9%) of 31 patients achieving a CR. After the full regimen of fludarabine and ^131^I-tositumomab, all 35 patients responded, with 30 patients (86%) achieving a CR. The 5-year estimated PFS rate was 60%. Baseline FLIPI was significantly associated (*p* = 0.003) with PFS. About 10 of 13 patients (77%) with baseline bone marrow Bcl-2 positivity demonstrated molecular remissions at 12 months. Toxicities were manageable and mainly hematological. Two of 35 patients (6%) developed human anti-murine antibodies (HAMA) after RIT. The authors concluded that this sequential treatment regimen was highly effective as a front-line therapy for FL, particularly for low- or intermediate-risk FLIPI patients. Zevalin^®^ also has been assessed as consolidation after Rituximab with short duration chemotherapy (49). Forty-one patients with previously untreated FL received rituximab for four consecutive weeks, followed by three cycles of rituximab combined with either CHOP (88%) or CVP (cyclophosphamide/vincristine/prednisone; 12%). To complete treatment, all patients received Zevalin^®^ 4–6 weeks after the final dose of chemotherapy. After completion of short-course rituximab/chemotherapy, 95% had objective responses, with 30% clinical CR. The clinical CR rate increased to 72% following RIT. After a median follow-up of 67 months, the estimated 5 year PFS and OS rates were 64 and 96%, respectively. Zevalin^®^ was well tolerated after short-course rituximab/chemotherapy and the authors concluded that a high CR rate and a long PFS were obtained using this scheme. Definitive demonstration of improved efficacy versus rituximab/chemotherapy alone requires a randomized study.

Radioimmunotherapy consolidation has been also assessed in other B-NHL subtypes and represents an especially relevant therapeutic alternative in elderly patients. In DLBCL patients, although the R-CHOP combination as standard regimen has led to improved outcomes, there is a group of poor-risk patients with a lower chance of being cured with standard R-CHOP, thus needing an alternative treatment strategy. Zinzani et al. published in 2010 the results of a phase II study assessing the efficacy and safety of Zevalin^®^ following four cycles of R-CHOP21, in 55 high-risk elderly (age ≥ 60 years) patients with previously untreated DLBCL (50). Forty-eight of the 55 patients received RIT. The OR rate for the entire treatment regimen was 80%, including 73% CR. About 8 of the 16 patients (50%) who achieved less than a CR after R-CHOP improved their remission status after RIT. With a median follow-up of 18 months, the 2-year PFS was estimated to be 85%, with a 2-year OS of 86%. RIT toxicity was relatively high, consisting of grade 3–4 neutropenia in 23 patients and thrombocytopenia in 15 patients. These results suggested that RIT could be considered as a promising alternative approach in high-risk elderly patients who are not candidates to SCT.

Recently, Smith et al. reported the results of a Phase II study of the Eastern Cooperative Oncology Group Study assessing R-CHOP in untreated MCL patients (51). The rational was based on three points: (a) MCL is predominantly a disease of patients older than 60 years of age, very often contraindicated for HD chemotherapy, (b) R-CHOP as initial therapy for untreated MCL had a high response rate, but remissions are not durable, and (c) RIT of MCL seemed to induce a high response rate but very short TTP (52). About 56 patients were eligible: 48 patients were treated with Zevalin^®^ standard dose whereas three received a 25% reduced-dose. The design required 52 eligible patients to detect a 50% improvement in the median time to treatment failure compared with that reported for six cycles of R-CHOP. For the 56 analyzed patients the overall response rate at completion of therapy was 82%: RIT improved quality of response in 22 patients: 16 patients converted from PR to CR/CRu, three patients from stable disease to CR/CRu, and three patients from stable disease to PR. With a median follow-up of 72 months, the median time to treatment failure was 34.2 months and the estimated 1.5 year time to treatment failure 69%. These results were better than those previously reported in patients treated by six cycles of R-CHOP and there was no unexpected toxicity after RIT.

Even if the potential of RIT as consolidation after R-CHOP induction therapy were to be confirmed in randomized large phase III studies, these different studies suggest that RIT is a relevant option as consolidation therapy in different subtypes of B-NHL, in order to decrease the number of chemotherapy courses in elderly patients or as an alternative of STC in high-risk patients.

## RIT Monotherapy in First-Line Treatment

Radioimmunotherapy can also be considered alone in front-line treatment. As shown by Emmanouilides et al., an injection of Zevalin^®^ earlier in the course of the disease leads to better outcomes (24). Indeed, the best results of non-myeloablative RIT administered alone (without chemotherapy) have been obtained as first-line treatment of FL (30). A single 1 week course of ^131^I-tositumomab therapy, as initial treatment, can induce prolonged clinical and molecular remissions. Seventy-six patients with stage III or IV FL received as initial therapy a single course of ^131^I-tositumomab therapy. Ninety-five percent of the patients responded, including 75% with a CR. The use of PCR to detect rearrangement of the bcl2 gene showed molecular responses in 80% of assessable patients who had a clinical CR. After a median follow-up of 5.1 years, the actuarial 5 year PFS for all patients was 59%, with a median PFS of 6.1 years. Of 57 patients who had a CR, 40 remained in remission for 4.3–7.7 years. Hematological toxicity was moderate, with no patient requiring transfusions or growth factors. No case of MDS has been observed. The immunization rate was higher than the rate observed in patients treated later in the course of disease.

Preliminary results of a European multicenter study using fractionated RIT Zevalin^®^ as a front-line therapy for patients with FL grade I–IIIa with at least one criteria of high tumor burden or B symptoms were presented at The American Society of Hematology in 2011 (53). Treatment consisted of two doses of Zevalin^®^ (11.1 MBq/kg) given 8–12 weeks apart. Patients with greater than 20% bone marrow involvement with lymphoma received four weekly infusions of Rituximab (375 mg/m^2^) and proceeded to fractionated RIT only if a repeat bone marrow biopsy demonstrated clearing of lymphoma with less than or equal to 20% involvement. 74 patients with a median age of 61 years (28–80), including 58 with stage III–IV disease were included. About 55 patients received the two-planned Zevalin^®^ infusion whereas 17 received only one infusion. At a median follow-up of 1.52 years (range 0.13–3.69 years) the PFS was 67%, 20/74 patients had progressed. The ORR was 97.1% with 64% of CR/CRu. The most common toxicity was hematologic and reversible. One case of MDS was diagnosed 26 months after treatment.

Recently, Scholz et al. also evaluated, in an international multicenter phase II clinical trial, the efficacy, and feasibility of Zevalin^®^ as first-line treatment for FL (54). Fifty-nine patients, median age 66 years (range, 51–83 years), were included. Treatment indication resulted from B symptoms, grade 3a, organ compression or infiltration, rapid growth and/or bulky disease. The ORR at 6 months after RIT was 87%, with 41% of the patients achieving CR, 15% CRu, and 31% PR. Median PFS was 25.9 months (95% CI, 18.2–33.7 months). RIT was well tolerated and the most common toxicity was hematologic and reversible. Patients with increased LDH may not benefit from RIT as much as patients with normal LDH do.

In these two studies assessing monotherapy with Zevalin^®^ in first-line treatment, the OR was superior to the response rate reported in chemotherapy-naive patients treated with four courses of rituximab monotherapy. Ghielmini et al. reported in this population a 67% response rate with only 9% CR in 64 patients receiving four courses of rituximab (55). RIT should be considered as an attractive therapeutic option in elderly patients or in patients with comorbidity.

## Fractionated RIT

The advantages of fractionated delivery of external radiation therapy may also apply to RIT (85). RIT produces a less uniform tumor dose distribution than external radiation therapy, because of the inability of MAbs to penetrate uniformly throughout the tumor, resulting in some tumor regions under-dosed. An absorbed dose heterogeneity ranging up to 400% was measured using quantitative autoradiography, in the Raji B-cell lymphoma animal model injected with anti-HLA-DR ^131^I-Lym-1 MAb (56). DeNardo et al. reported the following advantages for fractionated RIT: more uniform MAb distribution and radiation dose, patient-specific radionuclide and radiation dose, control toxicity by titration of an individual patient, reduced toxicity, increased injected activity, tumor radiation and efficacy, and prolongation of tumor response (57).

Several clinical studies have shown the benefits of fractionated RIT in patients with B-cell hemopathies. DeNardo et al. reported toxicity and efficacy of ^131^I-Lym-1 in 25 patients with relapsing NHL and five patients with chronic lymphocytic leukemia (58): 19/25 NHL patients had bone marrow involvement, with extensive marrow malignancy in seven cases, and all five patients with CLL had diffuse bone marrow infiltration. Patients were treated with doses of 30 or 60 mCi of ^131^I-Lym-1, 2–6 weeks apart; 11 of the 30 patients completed the intended 300 mCi. Treatment was interrupted by hematological toxicity in three patients and the development of HAMA in three; a 57% response rate was obtained. Based on this strategy, a dose escalation trial was designed to define the MTD of the first two of a maximum of four injections of ^131^I-Lym-1 given 4 weeks apart (58). ^131^I-Lym-1 was escalated from 40 to 100 mCi/m^2^. The non-myeloablative MTD for each of two doses of ^131^I-Lym-1 was 100 mCi/m^2^ in patients with less than 25% bone marrow involvement. All three entries in this patient cohort achieved CR.

The use of chimeric or humanized MAbs facilitates fractionation by reducing the immunization rate. Illidge et al. published the results of a protocol assessing four weekly infusions of 375 mg/m^2^ rituximab, followed by two fractions of ^131^I-rituximab, preceded by a 100-mg/m^2^ predose of rituximab, in relapsed indolent NHL (59). Induction therapy with rituximab significantly increased the effective half-life of ^131^I-rituximab, and high serum levels of rituximab after induction therapy correlated with increased effective half-life of the radiolabeled MAb. The pharmacokinetics of rituximab have previously been studied and found to be influenced by the availability of CD20 (the “antigen sink”), with the greater number of CD20-binding sites in bulky disease causing sequestration of rituximab and decreasing concentrations in the serum compartment. A negative correlation was observed between splenic and lymph node volumes and the rituximab serum levels after initial rituximab dosing. Interestingly, patients with large initial tumor burdens exhibited a greater than 40% increase in ^131^I-rituximab’s effective half-life between delivery of the first and the second fractions. The most likely explanation for these observations was the tumor responses observed clinically after the first therapeutic injection. The OR rate was 94%, with 50% CR. The median TTP was 20 months, significantly longer than for the last chemotherapy course. Moreover, fractionated ^131^I-rituximab provided cumulative whole-body doses of more than 120 cGy, approximately 60% greater than those obtained previously after a single course of RIT, without significant hematologic toxicity. These results demonstrated the high potential of fractionated injections for increasing RIT efficacy for NHL treatment.

## Pretargeted RIT of Lymphoma

Because of the long residence time of antibodies in the circulation and their efficient transfer to bone marrow, the DLT in RIT is hematological in most cases. The pretargeting approach that uses a low molecular weight as a carrier for radioactivity and unlabeled antibody conjugates, injected in advance, to target the radioactivity to tumors was geared at reducing non-tumor tissue exposure (60). Pretargeting may be achieved using a variety of methods. Two of them have been documented in large numbers of preclinical and clinical studies. One uses bispecific anti-tumor/anti-hapten antibodies to target low molecular weight radiolabeled haptens. The other uses antibody-avidin conjugates to target radiolabeled biotin derivatives. In both cases, improvements have been necessary to prevent excess circulating antibody conjugates to trap the radiolabeled molecule in the circulation. Antibody-avidin pretargeting thus generally includes a chase step before injection of the radiolabeled biotin derivative, while bispecific antibody pretargeting uses bivalent haptens that bind more tightly to bispecific antibody molecules attached to the target cell surface than to the excess bispecific antibody present in the circulation (60). With both approaches, tumor to non-tumor target activity uptake ratios are improved as compared to directly labeled antibodies and pretargeting was shown to be able to deliver tumoricidal irradiation doses. The feasibility of pretargeting in the clinic has been documented in several instances and it has been shown that it can increase OS in progressive, metastatic medullary thyroid carcinoma, a radioresistant and chemo-resistant solid tumor (61).

For lymphoma, and more generally in hematological diseases, it is believed that tumor cells are more readily accessible to radiolabeled antibodies and often more radiosensitive and, as a result, that sophisticated targeting approaches such as pretargeting are not required to achieve tumor control. Indeed, even though pretargeting approaches to lymphoma were proposed a long time ago in mice (62, 63), the literature remains poor in terms of reports of pretargeted RIT clinical trials of lymphoma. In one study of 10 patients with relapsed or refractory B-cell NHL, seven were treated with a streptavidin-conjugated anti-CD20 antibody (rituximab) to sequester yttrium-90-labeled DOTA-biotin (1.11 or 1.85 GBq/m^2^) after clearing excess streptavidin conjugate by an injection of biotin-N-acetyl-galactosamine (64). Radiolabeled biotin was shown to localize in the tumor, with rapid excretion of unbound radioactivity. Little non-hematologic toxicity was observed and hematological toxicity was limited and transient. Three complete and one PR were observed. However, humoral immune responses to streptavidin were demonstrated in 6 of 10 patients. In another phase I trial, a tetrameric single-chain anti-CD20-streptavidin fusion protein was used to target yttrium-90-labeled DOTA-biotin in NHL patients, again with a clearing step (65). In this study, the 15 patients received only 560 MBq/m^2^ of ^90^Y-DOTA-biotin that rapidly localized in tumor or was excreted in the urine. There were two complete remissions (90 and 325 days) and one PR (297 days) and hematologic toxicity was acceptable. However, patient immune responses against the antibody-streptavidin conjugate was frequent.

It is clear that the remarkable efficacy of directly labeled antibodies against lymphoma makes pretargeting less attractive here than in solid tumors. However, pretargeting could make activity escalation possible and could in principle achieve very high response rates without bone marrow ablation, as more recent preclinical studies suggest (66). Another perspective created by pretargeting is the possibility of using short-lived alpha-emitting radionuclides in consolidation therapy to eradicate MRD because of the fast tumor activity accretion provided by this technology (67).

## Target Antigens Other than CD20

The development of a RIT approaches against antigens other than CD20 targeted by rituximab appears relevant, especially if RIT is applied in combination with rituximab-based therapy, offering the possibility of targeting populations of cells not expressing CD20, or not responding to anti-CD20 cold MAbs. Several antigens have been tested: CD21, CD22, CD37, and HLA DR (68). Both radiolabeled anti-CD22 epratuzumab and anti-HLA-DR (Lym-1) MAb have shown efficacy in patients who have failed chemotherapy, either with low-grade or aggressive forms of NHL (58, 60, 69). CD22 is a transmembrane glycoprotein expressed on mature B-cells but not expressed on stem cells or plasma cells, and functions in B-cell regulation/activation. CD22 is highly expressed across malignant B-cell histologies. Epratuzumab, has good features for RIT because it is humanized, internalized by target cells, stably labeled using DOTA (1,4,7,10-tetraazacyclododecane-*N*,*N*′,*N*″,*N*″-tetraacetic acid), and administered without a loading dose of cold antibody, at variance with Zevalin^®^ or Bexxar^®^.

Sharkey et al. reported the results of a phase I/II trial assessing ^90^Y-epratuzumab in patients with relapsing B lymphoma (70). Patients had a pre-therapy imaging study with ^111^In-epratuzumab 1 week before ^90^Y-epratuzumab injection, starting at an activity level of 0.185 GBq/m^2^ in patients who had prior HD chemotherapy (Group 2), and at 0.370 GBq/m^2^ in patients who did not have a prior stem-cell transplantation (SCT) (Group 1), with escalation in 0.185 GBq/m^2^ increments. Radiation absorbed doses to liver, lungs, and kidneys averaged 0.55 ± 0.13, 0.28 ± 0.06, and 0.38 ± 0.07 mGy/MBq, respectively, with 0.14 ± 0.02 and 0.23 ± 0.04 mGy/MBq delivered to the whole-body and red marrow, respectively. Tumor doses ranged from 1.0 to 83 mGy/MBq for a 0.5-g lesion (median = 7.15 mGy/MBq). Anti-tumor effects were seen in both indolent and aggressive NHL. The data also suggest that anti-tumor responses of potentially equal magnitude can occur irrespective of tumor targeting and tumor size. Hence, tumor response did not correlate with the radiation dose delivered or with the tumor being visualized by external imaging. These observations should not been interpreted that there was a minor effect of targeting, but probably could be explained by the role of immunological mechanisms in tumor response after RIT. In this study, an immunization was detected in only two of 16 patients.

After demonstrating, in a single-center trial, the safety and preliminary efficacy of ^90^Y-epratuzumab administered at low 5 mCi/m^2^ (185 MBq/m^2^) doses repeated over several weeks (71), a multicenter phase I/II study was designed to assess fractionated ^90^Y-epratuzumab in NHL relapsing patients (72). Unlabeled epratuzumab was co-administered each week for a 1.5 mg/kg protein dose. The first week, ^111^In-epratuzumab was co-infused, with targeting of at least one known disease site by gamma-camera imaging 3–6 days later required to continue weekly ^90^Y-epratuzumab infusions. Patients with prior SCT underwent separate dose escalation, starting with 92.5 MBq/m^2^ weekly doses escalated in 2.5 mCi/m^2^ increments. Non-SCT patients started with 185 MBq/m^2^ weekly doses and 2.5 mCi/m^2^ increments that increased to 5.0 mCi/m^2^ to reach the highest dose level. Sixty-four patients (32 males, 32 females) were enrolled at five institutions in France and Germany between 2001 and 2007, including 35 FLs, 14 MCL, 11 DLBCL, and four patients with marginal zone lymphoma. These patients had one to five prior therapies (median: 2), including an anti-CD20-based therapy in 52 cases and a bone marrow transplant in 17 cases. The total ^90^Y treatment dose ranged from 0.185 to 1.665 GBq/m^2^, with comparable numbers treated at ≤0.37 (*N* = 17),>0.37–0.74 (*N* = 13),>0.74–1.11 (*N* = 16), and>1.11 GBq/m^2^ (*N* = 18). Even at the highest total ^90^Y dose of 1.665 MBq/m^2^ studied, grade 3–4 hematological toxicities were manageable with support in patients with<25% bone marrow involvement, and transient with 2–3 weeks median recovery to Grade 1. The overall OR rate was 62% (48% CR/CRu), including all NHL subtypes [FL: 74% (62%); MCL: 50% (21%); DLBCL: 30% (20%); marginal zone: 100% (100%)]; and in poor-risk patients [unresponsive to last therapy: 72% (56%); bulky disease: 60% (35%); elevated LDH: 56% (44%); positive bone marrow: 46% (31%); prior SCT: 41% (29%)]. For FL without prior SCT, response rates increased with total ^90^Y dose, with 92% CR/CRu at the highest dose levels (>1.11 MBq/m^2^). Patients with CR/CRu achieved long-lived responses continuing up to 5 years, including 24.6 month median PFS for 12 FL patients receiving>1.11 MBq/m^2^ total ^90^Y dose. This study demonstrated that fractionated RIT with ^90^Y-epratuzumab achieved high rates of durable CRs with manageable toxicity in previously treated lymphoma patients. While fractionation may have improved the diffusion of subsequent doses, the favorable efficacy and safety observed here even at HDs also could reflect antibody internalization with improved ^90^Y residence time, the more stable DOTA radio-labeling method used, or the lack of neutralizing antibodies induced with this humanized antibody.

Targeting of antigens other than CD20 appears particularly interesting in the context of consolidation therapy after rituximab-based therapy and the high injected activity achieved with fractionated ^90^Y-epratuzumab suggested the benefits of using this approach in DLBCL probably requiring higher dose than indolent lymphoma. A French phase II trial sponsored by the LYSA group assessed front-line treatment using fractionated RIT with ^90^Y-epratuzumab as consolidation therapy after courses of R-CHOP in previously untreated elderly (age>60 years) patients presenting with stage I/II bulky or stage III/IV DLBCL. Two infusions of ^90^Y-epratuzumab (2 × 555 MBq/m^2^, 7 days apart) were delivered 8 weeks after R-CHOP (73). Seventy-five patients have been accrued prospectively, 57 (76.0%) with Ann Arbor stage III/IV disease; 61/75 (81.2%) received the RIT. RIT toxicity consisted of grade 3–4 hematologic toxicity in 51/61 patients (83.6%). RIT’s severe non-hematologic toxicity consisted of grade-4 gastrointestinal in one patient (1.6%) and grade-4 infection in three (4.9%). Two patients (2.6%) developed MDS 5–20 months after RIT. The OR rate after 6 × R-CHOP14 was 94.6% (71/75); 52 patients (69.3%) achieved CR/CRu and 19 (25.3%) had a PR. In an intention-to-treat analysis, CR/CRu rate after 6 × R-CHOP14 followed by RIT was 72.0% (*N* = 54). At a median follow-up of 24 months (range 1–46), 18 patients experienced lymphoma progression and/or a related death, yielding an estimated 2 year EFS of 73.3% (60.7–82.5%) and an estimated 2 year OS of 83.2% (71.4–90.4%). For the 61 patients who received six courses of R-CHOP followed by ^90^Y-epratuzumab, ORR was 91.8% (56/61), 50 patients (81.9%) achieving CR/CRu. This phase II study clearly shows that fractionated RIT with ^90^Y-epratuzumab as a consolidation therapy after 6 × R-CHOP14 is feasible and tolerable in elderly untreated DLBCL patients with advanced disease. RIT improved response status observed after 6 × R-CHOP14. EFS data achieved with R-CHOP plus RIT compare favorably with those achieved with R-CHOP alone in the same patient population.

Another important perspective is the clinical evaluation of dual-targeted antibody/radioantibody therapy (74, 75). Combining an unconjugated anti-CD20 antibody therapy with a radioimmunoconjugate binding to a non-competing antigen might improve responses by allowing optimal uptake of each agent. Mattes et al. showed in an animal model the benefit of targeting a non-competing antigen for consolidation after RIT using ^90^Y-epratuzumab tetraxetan. Tumor response and survival rates were improved when a consolidation using anti-CD20 veltuzumab was delivered after anti-CD22 RIT (74). Moreover, Sharkey et al. (75) demonstrated that injection of cold MAb after the radioactivity dose provided higher efficacy than injection before RIT, and that the amount of predose of cold MAb should be minimized.

These preclinical data raise important questions, and a re-examination of RIT in the treatment of NHL was proposed by R. Sharkey, O. Press and D. Goldenberg in Blood in 2009 (86). The authors emphasized that in RIT clinical practice, nearly 900 mg of unlabeled anti-CD20 IgG antibody is predosed to the patient before the anti-CD20 ^90^Y or ^131^I RIT. Combining a naked anti-CD20 therapy with a radioimmunoconjugate binding to a non-competing antigen might improve responses by allowing optimal uptake of each agent. Preclinical models indicated that careful consideration should be given to predosing when using competing antibodies, but that consolidation anti-CD20 therapy enhances the efficacy of radioimmunoconjugate therapy.

## Optimization of Injected Activity Using Dosimetry

Pre-RIT dosimetry studies should allow the pharmacokinetics of each patient to be assessed and allow the injected activity to be adapted. Dosimetry studies performed before Bexxar^®^ injection showed large variability between patients, requiring injection of around 1.85–5.55 GBq of Bexxar^®^ to deliver a 75 cGy whole-body dose. Indeed, many patients are probably under-treated with the standard activity of 14.8 MBq/kg of Zevalin^®^. No dose-effect relationship has been clearly demonstrated in clinical studies, but patients had been heavily pre-treated. Dosimetry of RIT in first- line treatment should allow a better analysis of a dose-effect relationship.

Moreover, immuno-PET using iodine-124 or yttrium-86 could improve quantitative imaging and biodistribution analysis of Bexxar^®^ and Zevalin^®^, respectively. Preclinical studies in mice showed that ^89^Zr- and ^88^Y-Zevalin^®^ had a very similar biodistribution, implying that ^89^Zr-Zevalin^®^-PET might be well suited for prediction of ^90^Y-Zevalin^®^ biodistribution in a myeloablative setting (76).

In principle, dosimetry is useful to optimize radiation delivery during treatment in order to avoid toxicities to critical organs and to administer absorbed doses to tumors as highly as possible. Although firmly established in external radiotherapy treatment, this paradigm is not yet so settled in RIT. In NHL RIT, dosimetry studies are performed more to avoid hematological toxicities rather than to estimate absorbed doses to targets, which are usually disseminated throughout the patient and often of small sizes, particularly in residual diseases (77).

Dosimetry studies are mandatory with Bexxar^®^ treatments. Indeed, Wahl et al. showed that pharmacokinetics varied widely from one patient to another and more importantly that bone marrow toxicities were correlated to the total-body radiation dose (78). In contrast, no dosimetry study is required for Zevalin^®^ treatment as no toxicity/absorbed dose relationship was assessed during the initial multicenter study (79).

Since then, recent publications have described better toxicity/absorbed dose relationships. In a series of 58 FL patients treated by Zevalin^®^ RIT as a consolidation therapy after a first-line therapy, Delaloye et al. conducted a dosimetry study, assessing bone marrow absorbed dose using blood samples (80). The authors clearly found a correlation between whole-body and bone marrow absorbed doses with the PFS whereas no correlation was established between both absorbed doses and hematological toxicity. It is worth noticing that RIT was employed at an early stage of the therapeutic course as opposed to the initial phase I/II study where patients were heavily treated beforehand and probably suffered from major bone marrow impairments. This suggests that the sooner RIT is positioned along the therapeutic course, the better it is to assess an absorbed dose/effect relationship.

This assumption was also supported by a recent dosimetry study performed on 28 newly diagnosed FL patients treated by two fractions of Zevalin^®^ (81). Different bone marrow absorbed dose calculation approaches were compared to hematological toxicity. One main result was that blood samples were not able to predict hematological toxicities on patients even with low BM involvement (<20%) as opposed to image-based methods that clearly foresaw those toxicities. Another interesting result was that quantification based on 3D SPECT imaging afforded a better relationship than 2D whole-body imaging and echoed findings of He et al. (82). Indeed, in this last publication, 2D whole-body and SPECT imaging were also compared on 18 patients enrolled in clinical trial of HD myeloablative Zevalin^®^ therapy. The authors showed differences in residence time as high as 18% for the dose-limiting organ – the liver in this context.

This stresses an interesting point previously mentioned by Sjögreen-Gleisner et al. (83). In this recent review of lymphoma RIT dosimetry, the authors compared dosimetry results in light of the approaches used in different publications and concluded that there was a definite influence due to the dosimetry methodology used. This is not surprising of course, but does highlight the importance of standardization of dosimetry procedures, and advocates for the more widespread use of 3D SPECT/CT imaging which will lead to the more robust approach.

As already mentioned, a much more challenging task and goal would be to correlate tumor absorbed dose to therapeutic response. Dewaraja et al. also used SPECT/CT imaging to perform a dosimetry study on 20 NHL patients treated with Bexxar^®^ RIT (84). They found an improved tumor absorbed dose-response relationship once an equivalent uniform dose (EUB) that takes into account the biological effect of the cold antibody was considered. Publications showing absorbed dose-response/toxicity relationships are currently present even in NHL RIT therapy. In this context, dosimetry should be performed with a high quality level in order to demonstrate a correlation between absorbed dose-effect/toxicity relationships.

## Conclusion

Clinical results show that RIT has significant efficacy, but moderate response duration as a monotherapy in rituximab-refractory recurrence of NHL. A higher therapeutic impact may be achieved using RIT in HD myeloablative treatment, as consolidation after chemotherapy-immunotherapy, or as a first-line treatment. Randomized phase III clinical trials should be performed in naïve or minimally treated patients to better identify the benefits and the role of RIT in NHL in the era of rituximab based therapy. Dosimetry studies and fractionated administration could probably optimize the injected activity. Preclinical studies suggest the benefits of dual-targeted antibody/radioantibody therapy, combining an unconjugated anti-CD20 antibody therapy with a radioimmunoconjugate binding to a non-competing antigen, such as CD22.

## Conflict of Interest Statement

The authors declare that the research was conducted in the absence of any commercial or financial relationships that could be construed as a potential conflict of interest.

## Abbreviations

ADCC, antibody-dependent cell-mediated cytotoxicity; ASCT, autologous stem-cell transplantation; BEAM, carmustine, etoposide, cytarabine, melphalan; CDC, complement-dependent cytotoxicity; CR, complete response; DLBCL, diffuse large B-cells NHL; DLT, dose-limiting toxicity; DR, duration of response; EFS, event-free survival; FDG-PET, positron emission tomography with ^18^F-fluorodeoxyglucose; FL, follicular lymphoma; FLIPI, follicular lymphoma international prognostic index; GELF, Groupe d’Etude des lymphomes folliculaires; HAMA, human anti-murine antibodies; HD, high-dose; HD, high-dose; IPI, international prognostic index; MAb, monoclonal antibodies; MCL, mantle cell lymphoma; MDS, myelodysplastic syndrome; MRD, minimal residual disease; MRD, minimal residual disease; MTD, maximum tolerated dose; NHL, non-Hodgkin lymphoma; OR, objective response; OR, objective response; ORRs, overall response rates; OS, overall survival; PFS, progression-free survival; PR, partial response; R-CHOP, rituximab plus cyclophosphamide, doxorubicin, vincristine, prednisone; RIC, reduced-intensity conditioning; RIT, radioimmunotherapy; RIT-N, the international radioimmunotherapy network; SCT, stem-cell transplantation; TBI, total-body irradiation; TTP, time to progression; WHO, World Health Organization.
